# A Case of Tension Subcutaneous Emphysema Treated With Minimally Invasive Open-Window Thoracostomy Using a Wound Protector/Retractor and Three-Sided Taping

**DOI:** 10.7759/cureus.59920

**Published:** 2024-05-08

**Authors:** Hiroshi Mizuuchi, Tomoaki Masuno, Masahiro Hata, Kensaku Ito, Hidenori Kouso

**Affiliations:** 1 Department of Thoracic Surgery, Shimonoseki City Hospital, Shimonoseki, JPN; 2 Department of Respiratory Medicine, Oita Red Cross Hospital, Oita, JPN; 3 Department of Thoracic Surgery, Oita Red Cross Hospital, Oita, JPN

**Keywords:** vented chest seal, three-sided taping, wound protector/retractor, open window thoracostomy, minimally invasive surgical procedures, subcutaneous emphysema management

## Abstract

Subcutaneous emphysema is a common complication of thoracic surgery. Tension subcutaneous emphysema that causes airway obstruction is rare but life-threatening. This report presents a patient who developed tension subcutaneous emphysema after recurrent secondary pneumothorax surgery which was treated with minimally invasive open-window thoracostomy. A wound protector/retractor and three-sided taping were successfully used to prevent air from entering the subcutaneous space via the wound while draining trapped air without creating an open pneumothorax. This approach is an option for managing subcutaneous and intrathoracic air leakage in emergency situations.

## Introduction

Subcutaneous emphysema is a common complication of thoracic surgery, especially in patients with severe emphysema [1]. Progression of subcutaneous emphysema is rare but may cause potentially life-threatening airway obstruction [2,3]. This situation is known as tension subcutaneous emphysema [4]. Such an emergent situation requires a simple and straightforward treatment. This report demonstrates a case of tension subcutaneous emphysema after recurrent secondary pneumothorax surgery which was treated with minimally invasive open-window thoracostomy (OWT) using a wound protector/retractor and vented chest seal secured by three-sided taping.

## Case presentation

**Figure 1:**
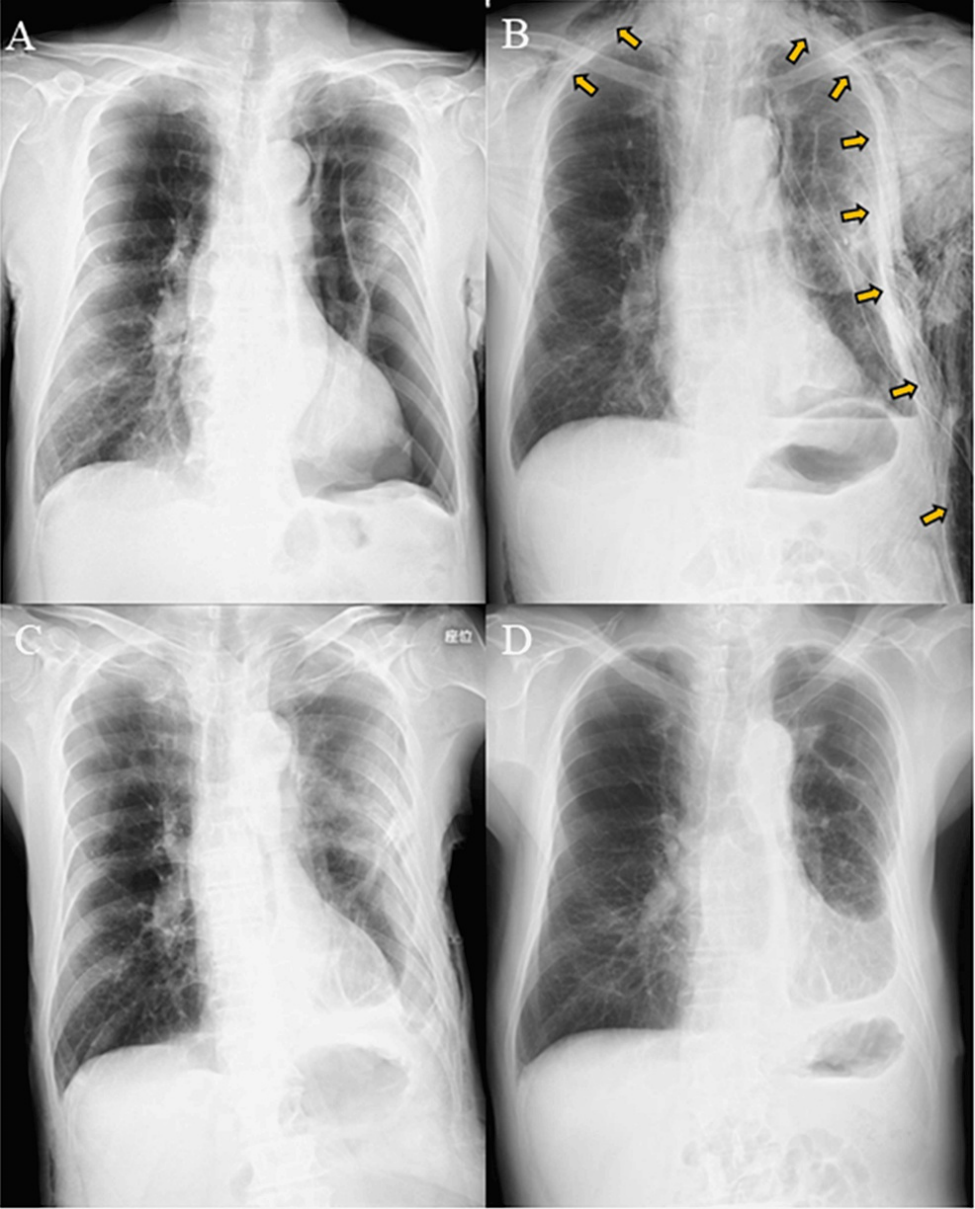
Chest radiography on (A) admission, (B) postoperative day 1, (C) day 9, and (D) day 30

**Figure 2:**
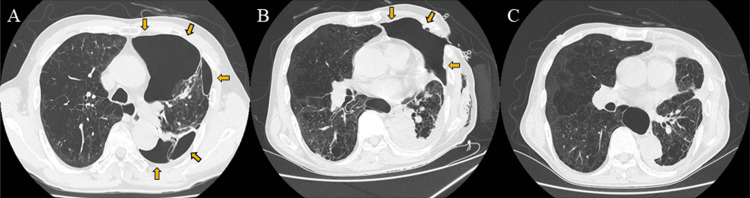
Chest computed tomography images (axial view) during hospitalization (A) Adhesion between the left upper lobe and chest wall, marked collapse of the left lower lobe, and diffuse emphysema were observed at admission. (B) Minimally invasive open-window thoracostomy using a wound protector/retractor in conjunction with three-sided taping improved the subcutaneous emphysema and resulted in left lower lung expansion. (C) Follow-up imaging showed left lung expansion and disappearance of the subcutaneous emphysema.

A 77-year-old man with a history of chemoradiation therapy for lung squamous cell carcinoma and inhalation therapy for chronic obstructive pulmonary disease for the past five years was transported by ambulance to the hospital because of respiratory distress. He had also undergone surgery for secondary pneumothorax caused by severe pulmonary emphysema two years before. Chest radiography revealed a collapse of the left lower lung and flattening of the diaphragm (Figure 1A). Computed tomography showed adhesions between the left upper lobe and chest wall, marked collapse of the left lower lobe, and diffuse emphysema (Figure 2A). After inserting a 24 Fr chest tube and connecting it to a digital chest drainage system, an air leak exceeding 5 L/minute was observed. Further intervention was warranted because of ongoing air leakage and insufficient lung expansion. In conjunction with the patient and his family, we decided to proceed with surgery under sedation and local anesthesia considering his age and overall condition. Informed consent for the procedure was obtained.

**Figure 3:**
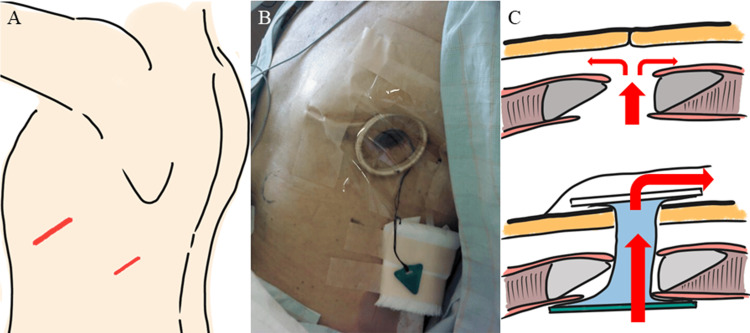
(A) Skin incisions were placed at the sixth and eighth intercostal spaces. (B) Photograph of the wound protector/retractor covered by three-sided taping. (C) Illustration depicting drainage of air from the thoracic cavity via the wound retractor and three-sided taping. Created by Dr. Hiroshi Mizuuchi

The patient was placed in the right lateral position after initiation of intravenous sedation. Skin incisions measuring 4 and 2 cm were made at the sixth and eighth intercostal spaces, respectively (Figure 3A). The site of the air leak was difficult to identify owing to extensive adhesions and respiratory movement of the hyperinflated lung. Two large bullous lesions were ligated in each of the left upper and lower lobes and two 24 Fr chest tubes were inserted. Although the air leak persisted, his respiratory condition remained stable. On postoperative day 1, significant subcutaneous emphysema developed, and he began to experience respiratory distress after a vigorous cough (Figure 1B). The sixth intercostal incision was reopened emergently, and the chest tube was removed, then an Alexis^®^ XS wound protector/retractor (Applied Medical, Santa Margarita, CA, USA) was applied to facilitate air drainage outside the thoracic cavity. The subcutaneous emphysema rapidly improved. Two days later, a vented chest seal was applied using three-side taping (Figure 3B) and supplemental oxygen was no longer required. By the next day, air leakage from the remaining chest drain had ceased and the drain was removed. On postoperative day 9, radiography and computed tomography confirmed expansion of the left lung (Figures 1C, 2B). Closure of the surgical wound under local anesthesia was performed on postoperative day 15 and he was discharged 15 days later. No recurrence of pneumothorax was observed (Figures 1D, 2C).

## Discussion

Postoperative subcutaneous emphysema occurs when air that is not draining via the chest tube enters the subcutaneous space through the surgical wound and infiltrates soft tissue in areas such as the chest, shoulders, neck, and face. When air leakage is extensive, which may occur in patients with emphysema or a bronchial stump fistula, conventional drainage methods may fail to prevent the progression of subcutaneous emphysema, which can cause tension subcutaneous emphysema with compromise of ventilation. In previous reports, subcutaneous emphysema has been treated using chest tubes [5], subcutaneous drains [6], and re-operation [1]. However, in an emergency situation, a rapid and simple bedside procedure is essential.

The procedure we describe involves a straightforward method of opening the surgical wound and inserting an Alexis^®^ wound protector/retractor, which can be considered a modified “blowhole” technique [7]. This approach provides a pathway for air to escape and prevents the entry of air into the subcutaneous space through the surgical wound, thanks to the device’s cylindrical membrane sheath and inner ring (Figure 3C). An OWT using a wound edge retractor is significantly less invasive than a conventional OWT, as it does not require bone resection [8].

However, OWT is associated with the risk of open pneumothorax. Therefore, OWT is generally considered appropriate for treating empyema after a bronchial stump fistula, where the lung and chest wall are fused [8,9]. In our patient, although some adhesions were present because of his previous surgery and radiation therapy for lung cancer, the left lower lobe had collapsed and his respiratory status was tenuous. We avoided open pneumothorax and stabilized his respiratory condition by applying a vented chest seal using three-sided taping (commercially available vented chest seals are not available in our hospital). A vented chest seal is generally useful for managing open pneumothorax caused by trauma [10]. To the best of our knowledge, this is the first report of its use in combination with OWT. This minimally invasive approach facilitates both thoracic drainage and lung expansion without inducing adhesions between the lung and chest wall.

## Conclusions

Tension subcutaneous emphysema after thoracic surgery is a rare but potentially life-threatening condition that necessitates immediate air drainage. Minimally invasive OWT using an Alexis^®^ wound protector/retractor (Applied Medical) and sealing the chest with three-sided taping proved advantageous in maintaining effective drainage and facilitating lung expansion. The simplicity and speed of opening the surgical wound and attaching the wound retractor are notable benefits of this approach. Fellow thoracic surgeons should consider this option for managing subcutaneous and intrathoracic air leakage in emergency situations such as tension subcutaneous emphysema.
